# Ultrasound-Assisted and Citric Acid-Guided Creation of ZnO Nanoparticles with Optimized Morphologies to Boost Malachite Green Photocatalysis

**DOI:** 10.3390/molecules30030466

**Published:** 2025-01-22

**Authors:** Xianlu Lei, Shuang Li, Jian Zeng, Meiqi Huang, Miaomiao Ma, Xueyan Ran, Xiang Chen, Yuting Yin, Qi Sun, Tao Le

**Affiliations:** Chongqing Collaborative Innovation Center for Rapid Detection of Food Quality and Safety, Chongqing Key Laboratory of Conservation and Utilization of Freshwater Fishes, Animal Biology Key Laboratory of Chongqing Education Commission, Chongqing Normal University, Chongqing 401331, China

**Keywords:** zinc oxide nanoparticles, morphological modulation, malachite green, simulated sunlight, photodegradation

## Abstract

Zinc oxide (ZnO) semiconductors are renowned for their cost-effective synthesis and superior catalytic attributes, making them prominent in environmental remediation applications. This study presents the synthesis of ZnO nanoparticles (NPs) with distinct morphologies, achieved by modulating citric acid concentrations in an ultrasonic-assisted hydrothermal process. The photocatalytic efficacy of these ZnO NPs in degrading malachite green (MG), a persistent environmental pollutant, was thoroughly investigated. Our findings reveal a strong correlation between the morphological features of ZnO catalysts and their photodegradation performance. Among the synthesized NPs, the chrysanthemum-shaped ZnO (denoted as USZ-0.1) demonstrated exceptional photocatalytic activity, attributed to its enhanced surface area and optimized nano-crystal aggregation. This structure facilitated the generation of a higher concentration of reactive oxygen species, leading to over 96.5% degradation of MG within 40 min under simulated sunlight in an acidic medium. This study underscores the potential of morphological manipulation in enhancing the photocatalytic properties of ZnO NPs for environmental applications.

## 1. Introduction

Malachite green (MG), a water-soluble cationic dye, is widely utilized as a colorant in leather, silk, and cotton industries, as well as a fungicide, ectoparasiticide, and disinfectant in aquaculture [1,2]. However, its widespread application is accompanied by significant environmental concerns due to its high stability, non-biodegradability, and strong resistance to light and oxidizing agents [3]. Furthermore, MG exhibits toxicity and mutagenicity toward humans, impacting the immune and reproductive systems [4]. It is highly toxic to mammalian cells at concentrations as low as 0.1 g/mL, causing respiratory and digestive tract inflammation upon inhalation or ingestion, respectively [5]. The persistence of MG residues not only directly harms the ecological environment but also poses a threat to human health through bioaccumulation [6,7]. Consequently, the development of efficient technologies to mitigate MG pollution is imperative for both ecological and human health protection.

Traditional methods for removing MG from the environment, such as adsorption [8], electrolysis [9], the electro-Fenton process [10], and biological treatment [11], have been employed. However, these methods often suffer from drawbacks, including limited efficacy, high energy consumption, the transfer of MG without degradation, and secondary contamination [12]. By contrast, photocatalytic degradation technology based on semiconductor materials has emerged as a promising alternative due to its low cost, high efficiency, and environmental friendliness [13].

Among the array of semiconductor photocatalysts, zinc oxide (ZnO) stands out as an ideal candidate for the degradation of organic pollutants due to its economic ease of preparation, excellent catalytic properties, wide band gap (~3.37 eV), and large exciton binding energy (60 meV) [14]. ZnO is globally recognized as a safe material with a substantial annual output, ranking third among commercial semiconductors [15]. The production cost of ZnO is only one-fourth that of titanium dioxide (TiO_2_), yet it has demonstrated better photocatalytic activity than TiO_2_ P25 under UV light for the removal of organic pollutants [16]. This can be attributed to the abundance of natural defect sites and a more efficient generation of reactive oxygen species on the surface of ZnO [17]. Despite its potential, the wide bandgap of ZnO leads to rapid recombination of electron-hole pairs, limiting its photocatalytic efficiency [18]. To address this challenge, researchers have explored various modification techniques, such as doping [19] and heterojunction formation [20], with a particular focus on morphology control [21], which has proven effective in enhancing charge separation and subsequently improving photocatalytic performance.

In this study, we aimed to synthesize ZnO nanoparticles (ZnO NPs) with different crystal morphologies via an ultrasound-assisted hydrothermal method by varying the amount of citric acid added. Our primary goal was to investigate the photocatalytic degradation of MG using these ZnO NPs and elucidate the relationship between the morphological characteristics of ZnO and its photocatalytic activity. We hypothesized that by modulating the morphology of ZnO NPs, we could significantly enhance their photocatalytic efficiency for MG degradation. To our understanding, this study marks a pioneering effort to comprehensively investigate the influence of ZnO morphology on its photocatalytic efficacy toward MG degradation, thereby advancing the development of ZnO-based photocatalysts for environmental remediation.

## 2. Results and Discussion

### 2.1. Characterizations of the As-Synthesized ZnO NPs

**Figure 1:**
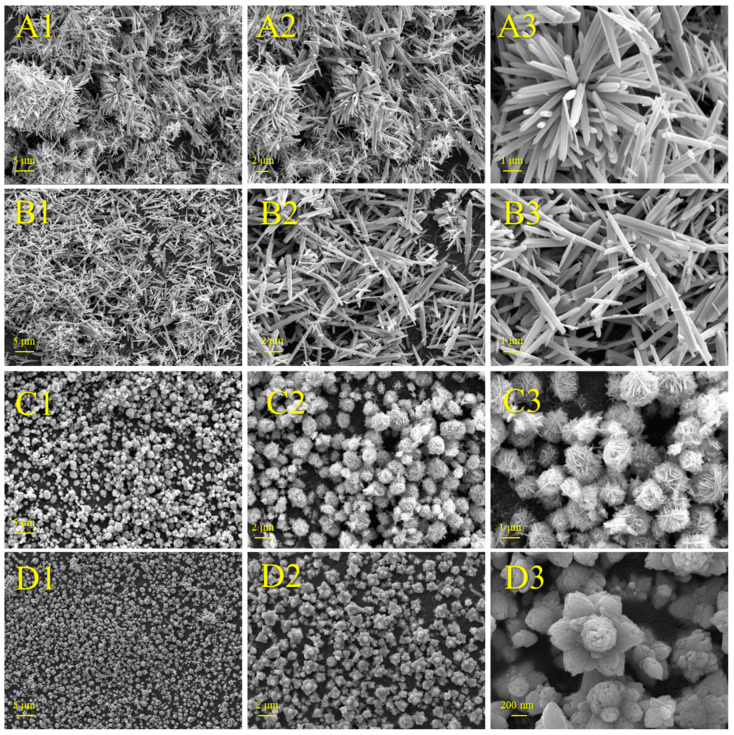
Scanning electron microscopy (SEM) images revealing the morphological diversity of ZnO NPs influenced by citric acid concentration: (**A**) hexagonal prism structure of USZ-0 (**A1**, **A2** and **A3** are low, medium and high magnification respectively); (**B**) chrysanthemum-like flower structure of USZ-0.1 (**B1**, **B2** and **B3** are low, medium and high magnification respectively); (**C**) self-assembled nanosheet petals of USZ-0.5 (**C1**, **C2** and **C3** are low, medium and high magnification respectively); (**D**) conical petal arrangement of USZ-1 (**D1**, **D2** and **D3** are low, medium and high magnification respectively).

The SEM images presented in Figure 1 provide a comprehensive analysis of the morphological characteristics of ZnO NPs synthesized via an ultrasound-assisted hydrothermal method, with varying quantities of citric acid as a modulator. The SEM images are categorized into four distinct groups, corresponding to the samples USZ-0, USZ-0.1, USZ-0.5, and USZ-1, which represent the synthesis without citric acid and with citric acid quantities of 0.1 g, 0.5 g, and 1.0 g, respectively. The SEM images reveal that the citric acid content has a profound impact on the morphology of the ZnO NPs. The USZ-0 sample, synthesized in the absence of citric acid, exhibits a classic hexagonal prism structure, with nanorods that are several micrometers in length and approximately 100 nm in diameter (Figure 1(A1–A3)). This morphology is indicative of the standard growth pattern of ZnO crystals under hydrothermal conditions without the influence of citric acid.

By contrast, the introduction of citric acid leads to a significant transformation in the ZnO NPs’ morphology. The USZ-0.1 sample showcases a three-dimensional flower-like structure, where the nanorods, measuring approximately 3.2 μm in length and 55 nm in diameter (Figure 1(B1–B3)), radiate outward from the center, resembling the petals of a flower. This structural evolution suggests that a low concentration of citric acid promotes the radial growth of nanorods, contributing to the formation of a flower-like architecture.

Further increasing the citric acid concentration to 0.5 g in the USZ-0.5 sample results in a dramatic change in the morphology. The SEM images (Figure 1(C1–C3)) depict a three-dimensional structure composed of numerous self-assembled nanosheets, forming a petal-like shape. This transition from nanorods to nanosheets indicates that higher citric acid concentrations facilitate the formation of a more complex, layered structure, which is essential for enhancing the photocatalytic properties of the material.

The USZ-1 sample, synthesized with the highest citric acid concentration of 1.0 g, presents a flower-like structure with multiple conical petals, each having an average diameter of around 760 nm (Figure 1(D1–D3)). The conical shape of the petals, as opposed to the nanorods in the USZ-0.1 sample, suggests that the increased citric acid concentration not only affects the size but also the shape of the individual components of the flower-like structure. The electron microscopy results demonstrate that the citric acid content, during the ultrasound-assisted hydrothermal process, is a critical parameter that governs the final morphology of the ZnO sample, which is crucial for optimizing its photocatalytic performance.

**Figure 2:**
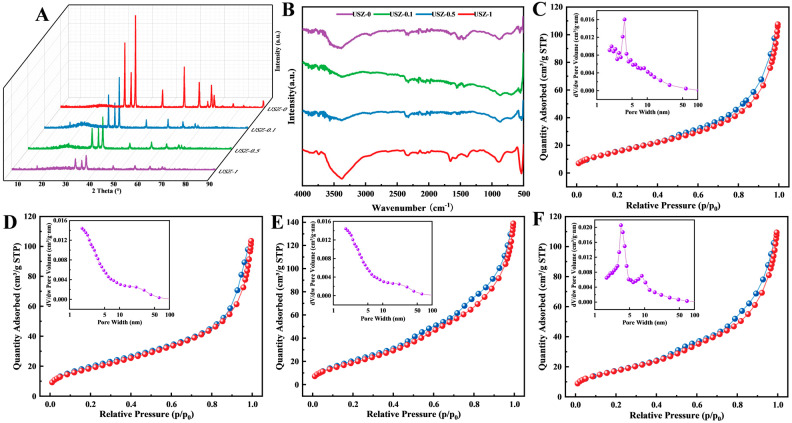
Characterization of USZ-x (x = 0.0, 0.1, 0.5, 1.0): (**A**) XRD patterns of USZ-x; (**B**) FT−IR spectra of USZ-x; (**C**–**F**) Nitrogen adsorption–desorption isotherms and BJH desorption pore size distribution for USZ-x, where red curves represent adsorption and blue curves represent desorption.

The XRD analysis (Figure 2A) confirmed the hexagonal wurtzite structure of the ZnO samples. The observed diffraction peaks at approximately 2θ = 31.8°, 34.4°, 36.2°, 47.5°, 56.5°, 62.8°, and 66.4° are attributed to the (100), (002), (101), (102), (110), (103), and (200) crystal planes, respectively, in accordance with JCPDS card number 36-1451 [22]. The pronounced diffraction peaks and the absence of extraneous peaks in the XRD patterns signify the high crystallinity and purity of the ZnO NPs. Notably, the XRD peak intensities diminish progressively with increasing citric acid content, suggesting a decrease in crystallinity due to the deformation of crystal structures.

The FT-IR spectra (Figure 2B) of the ZnO NPs display strong, broad absorption bands centered at approximately 3450 cm^−1^, indicative of O-H stretching vibrations. The peak at around 2360 cm^−1^ corresponds to C-O stretching vibrations, while the peak at 1637 cm^−1^ is associated with the bending vibration of hydroxyl groups. Characteristic bands at 563 cm^−1^, 881 cm^−1^, and 714 cm^−1^ were identified, corresponding to the stretching and deformation vibrations of Zn-O bonds, respectively [23,24].

The surface characteristics of the ZnO NPs were further elucidated through the Brunauer–Emmett–Teller (BET) gas adsorption system. Figure 2C–F present the nitrogen adsorption–desorption isotherms for four different ZnO nanocatalysts, with the inset in the upper left corner of each figure showing the Barrett–Joyner–Halenda (BJH) pore size distribution diagram. The isotherm analysis revealed that all ZnO samples exhibit type IV behavior according to the IUPAC classification, signifying the presence of mesoporous structures [25]. The BJH model analysis of the desorption data demonstrated variations in pore sizes among the prepared ZnO NPs. Specifically, the average BJH adsorption pore size for USZ-0.1 was determined to be 8.73 nm, which is significantly smaller than the average pore sizes for USZ-0 (10.40 nm), USZ-0.5 (10.75 nm), and USZ-1 (10.01 nm).

### 2.2. Photocatalytic Activity

The photocatalytic performance of the synthesized ZnO catalysts was assessed by monitoring the degradation of malachite green (MG) in aqueous solutions under different lighting conditions. The degradation was quantified by measuring the absorbance at 618 nm as a function of irradiation time.

**Figure 3:**
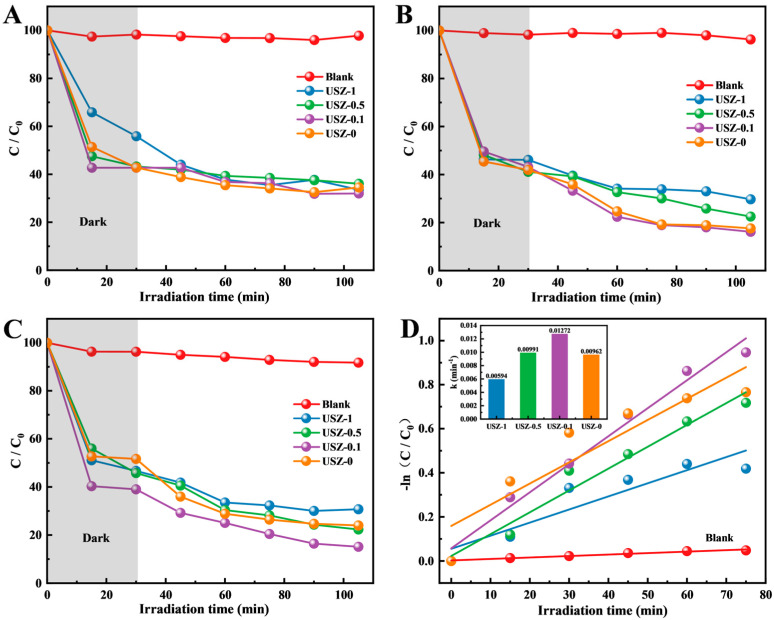
The degradation efficacy of USZ-x catalysts on malachite green (MG) solutions under various lighting conditions: (**A**) visible light, (**B**) ultraviolet (UV) light, and (**C**) simulated sunlight. (**D**) Photocatalytic degradation kinetic curves of MG solution catalyzed by USZ-x particles under simulated sunlight. The treatment in the absence of ZnO NPs served as the blank control group.

Under visible light illumination (λ ≥ 420 nm), the photocatalytic degradation of MG was initially evaluated. As depicted in Figure 3A, after 15 min of exposure, all ZnO catalysts demonstrated comparable degradation efficiencies, ranging from 56.0% to 61.2%. This indicates that the flower-like ZnO structures are effective in facilitating the photocatalytic process under visible light, with the morphological differences having a minimal impact on the initial degradation rates. Switching to UV light, the photocatalytic degradation efficiencies were more pronounced. After 30 min, USZ-0 and USZ-0.1 exhibited significantly higher degradation efficiencies of 77.3% and 77.6%, respectively, compared to USZ-0.5 and USZ-1 (Figure 3B). This suggests that the structural features of ZnO, particularly those influenced by citric acid content, play a crucial role in enhancing photocatalytic activity under UV light. The photocatalytic degradation under simulated sunlight showed a more distinct separation in efficiencies among the ZnO catalysts. After 60 min, USZ-0.1 achieved the highest degradation efficiency of 83.6%, outperforming USZ-0 (75.3%), USZ-0.5 (75.7%), and USZ-1 (69.9%) (Figure 3C). This trend underscores the influence of ZnO morphology on photocatalytic performance under solar-like conditions.

The degradation process under simulated sunlight was found to adhere to a pseudo-first-order kinetic model. The rate constants (k) for the blank group and the ZnO catalysts were calculated, with the blank group having a negligible k-value of 0.00023 min^−1^, indicating that self-photolysis is not a significant factor in MG degradation. The USZ-0.1 catalyst displayed the most remarkable photocatalytic reaction rate with a k-value of 0.01272 min^−1^, which is approximately 1.3 times that of USZ-0 (0.00962 min^−1^) and USZ-0.5 (0.00991 min^−1^) and 2.2 times that of USZ-1 (0.00594 min^−1^) (Figure 3D). This highlights the superior photocatalytic performance of the ZnO nanocatalyst prepared with an additional 0.1 g of citric acid under simulated sunlight conditions.

### 2.3. Optimization of Photodegradation Conditions

**Figure 4:**
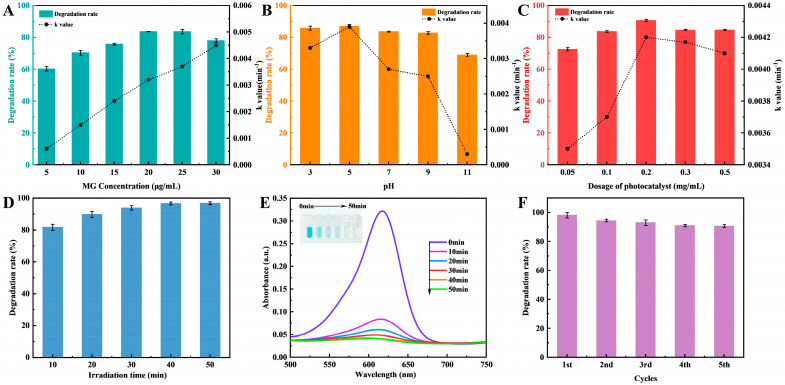
Optimization of photocatalytic conditions for USZ-0.1 in the degradation of malachite green (MG) under sunlight irradiation: (**A**) Influence of initial MG concentration; (**B**) effect of solution pH; (**C**) impact of photocatalyst dosage. (**D**) Degradation rate and (**E**) UV–vis absorption spectra of MG solution at various time intervals under the optimized experimental conditions: [MG]_0_ = 20 μg/mL, pH = 5, [USZ-0.1] = 0.2 mg/mL. (**F**) Recycling efficiency assessment of USZ-0.1 in the photocatalytic degradation of MG.

The photocatalytic degradation efficiency of MG was optimized by methodically adjusting the reaction conditions for the use of the USZ-0.1 catalyst. The impact of MG concentration on degradation efficiency was examined, revealing a peak efficiency of 83.7% at a concentration of 20 μg/mL, followed by a slight decline (Figure 4A). This trend suggests that while higher concentrations provide more reactant molecules for degradation, excessively high concentrations may lead to adsorption saturation or aggregation, which can hinder the photocatalytic process [26].

The pH of the solution was identified as a critical factor affecting the photocatalytic reaction [27]. The degradation efficiency was maximized at a pH of 5.0, reaching 87.2% (Figure 4B). This is attributed to the pH-dependent charge distribution on the ZnO NPs surface, which influences the generation and migration of photoinduced electron-hole pairs, thereby affecting photocatalytic activity [28,29].

The dosage of the USZ-0.1 catalyst was also optimized, with the highest degradation efficiency of 90.6% observed at a dosage of 0.20 mg/mL (Figure 4C). The initial increase in efficiency with catalyst dosage is due to the increased number of active sites available for reaction [30]. However, beyond the optimal dosage, the light screening effect and potential for catalyst agglomeration and precipitation can reduce the degradation efficiency [31]. The reaction time was optimized under the best conditions, showing a gradual increase in solar degradation efficiency, with nearly complete degradation of MG after 40 min, exceeding 96.5% efficiency (Figure 4D). The change in UV–vis absorption spectra of MG over time further confirmed these findings (Figure 4E), as the characteristic peaks of MG diminished, indicating degradation.

The stability and recyclability of the USZ-0.1 catalyst were assessed through cyclic degradation experiments. After five cycles, the catalyst maintained 92.4% of its initial degradation efficiency (Figure 4F). The slight decrease in efficiency can be attributed to minor catalyst loss during the cycling process. These results demonstrate the robust stability and recyclability of the USZ-0.1 catalyst, making it a promising candidate for practical applications in MG photodegradation.

The data encapsulated in Table 1 reveal that the ZnO NPs synthesized in this study exhibit superior photocatalytic efficiency. Specifically, our ZnO catalyst achieved a remarkable 96.67% degradation of MG within a mere 50 min under simulated sunlight conditions. This efficiency is notably higher than that reported for other catalysts, such as SnO_2_/ZnO, which required 150 min to achieve 98% degradation under visible light [24], and CdS, which, despite its high efficiency under UV light, necessitated a shorter irradiation time but at the cost of a higher catalyst dosage [32].

### 2.4. Photodegradation Mechanism

The photocatalytic journey of MG begins with the illumination of ZnO NPs, which, when subjected to light of sufficient energy, facilitates the excitation of electrons from the valence band (VB) to the conduction band (CB). This transition leaves behind holes (h^+^) in the VB and releases photogenerated electrons in the CB, as depicted in Equation (1). The holes then engage with water molecules or hydroxide ions (OH^−^) to form highly reactive hydroxyl radicals (OH·), as shown in Equations (2) and (3). Concurrently, the electrons in the CB react with molecular oxygen to produce superoxide radicals (O_2_^⋅−^), as per Equation (4).

ZnO + hv → h^+^ + e^−^ (1)

h^+^ + H_2_O → OH·+ H^+^ (2)

h^+^ + OH^−^ → OH· (3)

e^−^ + O_2_ → O_2_^⋅−^ (4)

O_2_^⋅−^ + ⋅OH· + MG → CO_2_ + H_2_O (5)

**Figure 5:**
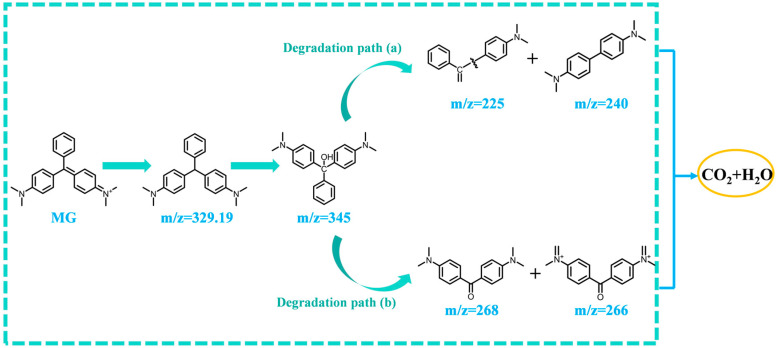
Possible pathway for USZ-0.1 for photocatalytic degradation of MG.

These reactive radicals, OH· and O_2_^⋅−^, are the principal agents in the oxidative breakdown of MG. As illustrated in Figure 5, the primary metabolite of MG, leucomalachite green [35], with a mass-to-charge ratio (*m*/*z*) of 329.19, is attacked by OH· radicals, leading to the formation of tri-phenyl methanol (*m*/*z* = 345). This intermediate can follow two distinct decay pathways: Pathway (a) results in the formation of a benzophenone intermediate (*m*/*z* = 225) and 4,4′-dimethyl-amino biphenyl (*m*/*z* = 240), while Pathway (b) yields two benzophenone derivatives (*m*/*z* = 268 and 266) [36,37]. Ultimately, these intermediates are completely mineralized into carbon dioxide (CO_2_) and water (H_2_O) [38], as described in Equation (5). It is important to note that while the intermediate products, such as *m*/*z* 268 and *m*/*z* 266, were not directly detected in our reaction mixture using mass spectrometry (MS), their presence was inferred on the basis of the established photocatalytic degradation pathways of malachite green (MG) reported in the literature [35,36,37]. Our experimental observations, which include the gradual weakening of the characteristic absorption peaks of MG, are consistent with the effective degradation of MG and support the plausibility of these intermediate products. The degradation pathways proposed in our study are thus based on a combination of literature-supported mechanisms and experimental evidence, providing a reasonable explanation for the observed photocatalytic degradation process.

The morphology of ZnO NPs plays a pivotal role in the photocatalytic process. Non-porous structures like ZnO NPs ensure that reactions predominantly occur on the crystal surface. The transformation of rod-shaped ZnO into flower-like structures through the addition of citric acid during ultrasonic-assisted hydrothermal synthesis enhances the specific surface area, which is crucial for the generation of free radicals and the efficiency of photocatalytic degradation. However, the relationship between surface area and degradation efficiency is not linear. The chrysanthemum-shaped USZ-0.1, with a specific surface area of 74.57 m^2^/g, exhibits higher degradation efficiency for MG compared to the hydrangea-shaped USZ-0.5 (68.28 m^2^/g), despite its smaller surface area. Conversely, the narcissus-like USZ-1.0, with a surface area of 63.20 m^2^/g, shows the lowest degradation efficiency, which may be due to its high degree of aggregation. This aggregation can impede light penetration and reduce the availability of sites for photocatalytic reactions [39], suggesting that a balance in the degree of aggregation is essential for optimal photocatalytic performance. Consequently, the photocatalytic degradation of MG by ZnO NPs is a multifaceted process that involves the generation of reactive radicals and the transformation of MG into less harmful byproducts. The morphology of the ZnO NPs, particularly the flower-like structures, significantly influences the photocatalytic efficiency, highlighting the importance of structural optimization in the design of photocatalytic materials.

## 3. Experiments

### 3.1. Synthesis of ZnO NPs with Varying Floral Structures

Zinc acetate dihydrate [Zn(CH_3_COO)_2_·2H_2_O], sodium hydroxide (NaOH), citric acid (C_6_H_8_O_7_), and malachite green (MG) were sourced from Aladdin Biochemical Technology Co., Ltd. (Shanghai, China). All other reagents used in this experiment were of analytical grade and used without further purification.

**Scheme 1:**
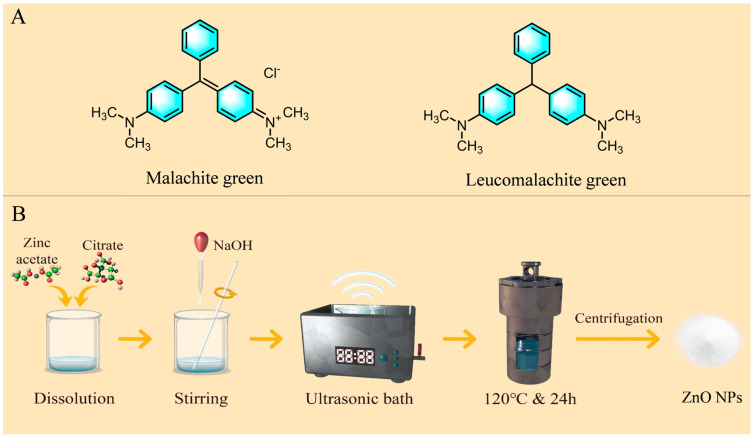
(**A**) Chemical structural formulae of malachite green (MG) and leucomalachite green; (**B**) flow chart of ultrasound-assisted and citric acid-guided synthesis of zinc oxide nanoparticles (ZnO NPs).

The preparation of ZnO nanoparticles (ZnO NPs) and the adjustment of their floral structures were carried out according to a modified version of the established method [40]. As illustrated in Scheme 1, the procedure involved weighing 0.2 g of zinc acetate dihydrate and varying amounts of citric acid (0.0 g, 0.1 g, 0.5 g, and 1.0 g), which were then dissolved in 60 mL of distilled water. Subsequently, 20 mL of 1 mol/L sodium hydroxide solution was added dropwise to the solution, followed by ultrasonication at room temperature for 1 h. The reaction mixture was then transferred to a 100 mL polytetrafluoroethylene-sealed autoclave for a hydrothermal reaction at 120 °C for 24 h. The resulting products were centrifuged, washed, and dried to yield four types of ZnO NPs, denoted as USZ-x (x = 0.0, 0.1, 0.5, 1.0), corresponding to the different amounts of citric acid used in the synthesis process.

### 3.2. Characterizations

The morphological structure of the synthesized ZnO NPs was initially examined using an SU-3500 scanning electron microscope (SEM, Hitachi, Japan). Subsequently, the crystal properties of the samples were analyzed using a Rigaku D/Max-2400 X-ray diffractometer (XRD, Rigaku, Japan) operated at 50 mA and 30 kV, with Cukα as the radiation source. Fourier transform infrared (FTIR) spectra were collected on a Nicolet-iS10 FTIR instrument (Thermo Scientific, Waltham, MA, USA) equipped with an attenuated total reflectance (ATR) accessory. N_2_ adsorption and desorption at 77 K were analyzed using an ASAP 2020 Plus HD88 specific surface and porosity analyzer (BET, Micromeritics, Norcross, GA, USA). The pore size distribution and total pore volume were determined utilizing the Barrett–Joyner–Halenda (BJH) method with support from the desorption branch of the N_2_ isotherm.

### 3.3. Photocatalytic Degradation of MG

Malachite green (MG) was employed as a model organic pollutant to assess the photocatalytic degradation activity of different ZnO NPs. In a typical photodegradation experiment, 10 mg of ZnO catalysts (0.2 mg/mL) were added to a 50 mL aqueous solution of MG (20 mg/L) in a quartz tube. The mixture was stirred in a photochemical reactor equipped with a circulating cooling system and 350 W xenon lamp (Nanjing Xujiang Instrument Co., Ltd., Nanjing, China) for 30 min in the dark to establish adsorption–desorption equilibrium. Following this, the Xe lamp was turned on, and the MG solution was irradiated with visible light, ultraviolet light, and simulated sunlight, respectively. At specified intervals of 15 min, 3 mL of the reaction solution was collected and centrifuged. The absorbance of the supernatant was measured at 618 nm (the maximum absorption wavelength of MG) using a UV–visible spectrophotometer. The MG degradation efficiency was calculated as the ratio of Ct/C0, where Ct and C0 represent the absorbance of the supernatant at different reaction times and the absorbance of the initial MG aqueous solution, respectively. A blank control group was included for all photocatalytic experiments. To evaluate the recyclability and stability of the catalysts, the ZnO NPs were recovered by centrifugation, cleaned, dried, and reused in subsequent photocatalytic degradation experiments.

### 3.4. Statistical Analysis

All experiments were performed in triplicate, and the results are reported as means ± standard deviations. Tukey’s test was used to identify significant differences in the results of the one-way analysis of variance (ANOVA). Data analysis was conducted using the statistical software package SPSS version 22.0 (SPSS Inc., Chicago, IL, USA).

## 4. Conclusions

The present investigation successfully synthesized ZnO NPs with diverse morphologies through an ultrasonic-assisted hydrothermal process, offering a viable strategy for enhancing the photocatalytic removal of organic pollutants. The citric acid concentration emerged as a critical parameter in shaping the ZnO nanocrystals’ morphology, which in turn significantly influenced the photocatalytic degradation of MG. The specific surface area, crystal dimensions, and aggregation degree of the ZnO catalysts were identified as key factors affecting their photocatalytic performance. Notably, the chrysanthemum-like USZ-0.1, synthesized with 0.1 g of citric acid, outperformed other morphologies in degrading MG, achieving a remarkable efficiency of over 96.5% under simulated sunlight within 40 min in an acidic environment. The USZ-0.1 catalyst also exhibited commendable recyclability and stability, highlighting its potential for practical environmental applications. This research provides valuable insights into the photocatalytic behavior of ZnO with varied morphologies and contributes to the broader understanding of semiconductor materials for the treatment of organic pollutants.

## Figures and Tables

**Table 1:** Comparison of the photocatalytic properties of different catalysts for MG.

Catalyzer	Catalyst Dosage (mg)	MG Concentration (mg/L)	Time (min)	Illumination Type	Efficiency (%)
SnO_2_/ZnO	50	10	150	Visible light	98 [24]
CdS	50	30	60	UV light	95 [32]
NiWO_4_	25	100	160	Visible light	80 [33]
WSe_2_/ZnO	25	2	120	Visible light	80 [34]
ZnO	25	25	50	Simulated sunlight	96.67 (This work)

## Data Availability

Data are contained within the article.
